# 

**Published:** 1913-04

**Authors:** 


PUBLISHED MONTHLY. ATLANTA SUBSCRIPTION $1.00

### JOURNAL-RECORD OF MEDICINE

Successor to { Atlanta Medical and Surgical Journal, Established 1855, and Southern Medical Recor[illegible], Established 1870. } 60th Yea[illegible]

Vol. LX. ATLANTA, GA., APRIL, 1913. No.

